# Effects of temperature, time, and solvent ratio on the extraction of phenolic compounds and the anti-radical activity of *Clinacanthus nutans* Lindau leaves by response surface methodology

**DOI:** 10.1186/s13065-017-0285-1

**Published:** 2017-06-14

**Authors:** Intan Soraya Che Sulaiman, Mahiran Basri, Hamid Reza Fard Masoumi, Wei Jian Chee, Siti Efliza Ashari, Maznah Ismail

**Affiliations:** 1 Nanodelivery Group, Department of Chemistry, Faculty of Science, Universiti Putra Malaysia, 43400 Serdang, Selangor Malaysia; 2 Laboratory of Molecular Biomedicine, Institute of Bioscience, Universiti Putra Malaysia, 43400 Serdang, Selangor Malaysia; 3 Department of Biomaterials, Iran Polymer and Petrochemical Institute, Tehran, Iran

**Keywords:** *C. nutans*, Central composite rotatable design (CCRD), Total phenolic content, 1,1-diphenyl-2-picrylhydrazyl (DPPH), Optimization, Anti-radical activity

## Abstract

**Background:**

*Clinacanthus nutans* Lindau is a well-known plant, native to tropical Asian countries. Reports on this plant that is rich in phenolic compounds have focused on its therapeutic anti-inflammatory, anti-herpes simplex, antioxidant, and anti-cancer characteristics. In this paper, the influence of the extraction parameters—temperatures (60–80 °C), times (80–120 min), and solvent ratios (70:30–90:10) of water:ethanol were investigated using response surface methodology in order to determine the optimum extraction conditions that could produce maximum extraction yields of the phenolic compounds and the highest anti-radical activity of the *C. nutans* extract.

**Results:**

The optimum conditions suggested by the predicted model were: an extraction temperature of 60 °C, an extraction time of 120 min and a water:ethanol solvent ratio of 90:10 v/v%. The residual standard error of 0.2% indicated that there was no significant difference between the actual and predicted values and it proved that the models were adequate to predict the relevant responses. All the independent variables had a significant effect (*p* < 0.05) on all the responses which indicated that all extraction parameters employed in this study were important in the optimization process. The R^2^ values for three responses, extraction yields, DPPH radical scavenging activity and TPC were 0.9999, 0.9999 and 0.9983 respectively, suggesting that the quadratic polynomial models developed were satisfactorily accurate to be used in analyzing the interactions of the parameters (response and independent variables).

**Conclusion:**

This study could be useful in the development of cosmeceutical products containing extracts of *C. nutans*.

## Background

*Clinacanthus nutans* Lindau (*C. nutans*) is a plant that is commonly known in Malaysia as Sabah Snake Grass, and is widely used in folk medicine. Native to tropical Asian countries such as Malaysia, Thailand and Singapore, *C. nutans* has traditionally been used as an herbal remedy for insect bites [1, 2], detoxification [3, 4], herpes zoster infections [5] and to reduce the progression of cancer [6]. Numerous reports have documented the biological activity of *C. nutans*, including its anti-viral [7–9], anti-inflammatory [10], antioxidant [11], antinociceptive [12], antiaging [13] and anti-cancer [14, 15] properties. Previous investigations have established the presence of various polyphenols such as vitexin, isovitexin, shaftoside, isomollupentin-7-*O*-beta-glucopyranoside, orientin, isoorientin, kaempferol, sinapic acid, vanillin, quercetin, rutin trihydrate, syringic acid, protocatechuic acid, 4-vinylphenol and 7-hydroxyflavone in the extracts of *C. nutans* leaves [14, 16–18]. The ethnomedicinal uses of the *C. nutans* plant, its chemical constituents and pharmacological properties associated to its therapeutic potential has been of much research focus [19–21]. Plant polyphenols have drawn increasing attention due to their potent antioxidant properties and their marked effects in the prevention of oxidative stresses [22, 23].

As plants survive in environments with massive exposure to ultraviolet radiation, they are perfect antioxidant sources due to their rich endogenous antioxidants [24]. In addition, most quality products formulated from nature-based ingredients have had excellent safety records in the marketplace, which has led to a growing interest in herbal formulations [24]. Due to their relative safety and wide acceptance, plant polyphenols have been incorporated into pharmaceuticals and cosmeceuticals as alternatives to synthetic antioxidants [25]. Moreover, antioxidants can enhance the biological functions of cells by virtue of their radical scavenging activities [26]. About 1.5–5% of our consumed oxygen is converted into reactive oxygen species (ROS). ROS are harmful free radicals that are constantly being produced as by-products in the electron transport chain of aerobic metabolism in the mitochondria [27]. The imbalanced production of ROS and anti-oxidative defense in the body can led to oxidative stress which can result in serious cell damage [28]. Plant polyphenols are an example of non-enzymatic antioxidants. They work by interrupting free radical chain reactions [29]. The antioxidant compounds react by binding to the free radicals, thus preventing them from reaching their biological target [29, 30]. As a result, polyphenols offer protection against various diseases which are caused by oxidative damage due to the harmful effects of ROS to the body [28].

Many factors can influence the efficiency of antioxidant phenolic extractions from the plant matrices. Due to the unstable nature of phenolic compounds, each phenolic source demands an individual approach for extraction and optimization [31]. No universal extraction technique is ideal due to the diversity of polyphenols [32]. Therefore, extraction conditions are important to maximize extraction yields and enrich the phenolic components. Several factors need to be considered when employing extraction techniques including the solvent types and ratios, extraction temperatures, extraction times, and solid to liquid ratios to ensure a complete extraction of the compounds of interest, while avoiding chemical modification [31, 33–35]. In practice, ethanol is often more preferred for food and pharmaceutical processing compared to other solvents due to its safety and affordability [36, 37]. Previous investigations established that extractions with binary solvents or aqueous alcoholic mixtures contributed to high antioxidant capacities [38]. This could be explained by the inability of ethanol to extract 100% of the phenolic compounds, some of which are more water-soluble (hydrophilic). Therefore, the presence of water in the extraction eases the release of hydrophilic antioxidants [38]. Reflux extraction is a simple, rapid, and economical technique for the extraction of antioxidant secondary metabolites from *C. nutans* which allows a better control of the extraction parameters such as extraction time, temperature and solvent ratio. Furthermore, extraction conditions play a critical role in pharmaceutical productions, especially for extracts that are produced in low yields [39].

Response surface methodology (RSM) is a systematic design for process development and optimization. It helps in evaluating the relative significance of variables that influence the process [40]. RSM is widely used to overcome classical optimization limitations which is time consuming, expensive and lacks data evaluation [41, 42].

There are no known optimization studies on the extraction of antioxidant compounds from *C. nutans* leaves. The objective of this study is to optimize the extraction conditions (extraction temperature, extraction time, and solvent ratio) needed to extract the phenolic components in *C. nutans* leaves and to determine the optimum conditions for the maximum extraction yields and the highest anti-radical activity of the extracts.

## Methods

### Materials

All the chemicals and reagents used were of analytical grade. Ethanol, 1,1-diphenyl-2-picrylhydrazyl (DPPH) and Folin–Ciocalteu phenol reagents were obtained from Sigma-Aldrich (Germany). Sodium carbonate (Na_2_CO_3_) was purchased from Merck (Darmstadt, Germany). Distilled water was purified in our laboratory.

### Plant material

Fresh leaves of *C. nutans* were collected from a botanical farm in Jelebu, Negeri Sembilan, Malaysia in January 2014. The plant was authenticated by biologist Associate Prof. Dr. Rusea Go and the specimen voucher (RG5125) was deposited at the Herbarium Unit of Universiti Putra Malaysia.

### Extraction

Fresh leaves of *C. nutans* were air-dried in the shade and ground to a fine powder. The finely-powdered *C. nutans* (20 g) was placed in a conical flask and mixed with an extraction solution. The extraction was performed at a solid to liquid ratio of 1:10 (w/v) in a reflux system with a magnetic stirrer and a temperature-controlled water bath. All the experiments were performed in triplicate. After the reflux extraction, the samples were filtered, and concentrated using a rotary evaporator (Rotavapor R-210, Buchi, Switzerland) at approximately 60 °C, weighed and stored at −20 °C prior to further analysis.

### Free radical scavenging activity (DPPH assay)

Radical scavenging activity was performed according to the protocol by Ramadan et al. [43]. A 0.2 mM methanolic solution of 1,1-diphenyl-2-picrylhydrazyl (DPPH) was freshly prepared. Initially, 0.6 ml of sample (2000 ppm) was mixed with 2.34 mL of DPPH solution. After being vortexed for 20 s, the resulting mixture was allowed to stand for 30 min in the dark. The UV–Visible absorbances of the reaction mixture were recorded at 515 nm using a spectrophotometer (Shimadzu UV-1601). Trolox was used as a standard and the DPPH scavenging activity of *C. nutans* extracts was expressed as an inhibition percentage. The inhibition percentage was calculated according to the following equation.

1 $$\% {\text{Inhibition}} = \frac{{({\text{Absorbance of control}} - {\text{Absorbance of sample}})}}{\text{Absorbance of control}} \times 100$$

### Determination of the total phenolic content (TPC)

The TPC of *C. nutans* extracts was determined according to Negi [44]. 0.5 mL of the sample was prepared in methanol and mixed with 2.5 mL of diluted Folin–Ciocalteu’s reagent (tenfold). 2 mL of 7.5% of Na_2_CO_3_ was added. The mixture was allowed to stand for 30 min at room temperature before the absorbance was measured at 760 nm using a UV–Visible spectrometer (Shimadzu UV-1601).

### Experimental design for the response surface procedure

A three-factor-five level central composite rotatable design (CCRD) was employed to determine the optimum extraction conditions of the *C. nutans* leaves. The independent variables selected in this study were extraction temperature (°C), extraction time (min) and solvent ratio (water: ethanol) (v/v%) toward the responses; extraction yield (weight %), DPPH radical scavenging activity (inhibition %) and total phenolic content (mg gallic acid equivalent/g extract). A total of 20 experiments were generated using the Design Expert^®^ software (Version 7, Stat. Ease Inc., Minneapolis, USA). Experiments with three independent variables consisting of eight factorial points, six axial, and six center points were carried out. Experiments were run randomly in order to minimize the effects of unexplained variability in the actual responses due to extraneous factors [45]. A summary of the independent variables and their coded levels are shown in Table 1.

**Table 1:** Coded independent variables used in CCRD design

Symbol	Independent variables	Coded level				
		−1.68	−1	0	+1	+1.68
A	Extraction temperature (°C)	53.18	60.00	70.00	80.00	86.82
B	Extraction time (min)	66.36	80.00	100.00	120.00	133.64
C	Solvent ratio (water: ethanol), v/v%	63.18:36.82	70:30	80:20	90:10	96.82:3.18

### Statistical analysis

Analysis of variance (ANOVA) was performed to determine the significant differences between the independent variables. Reduced model (*p* < 0.05) and multiple regressions were employed in analyzing the experimental data. The design was expressed by polynomial regression as shown in Eq. 2.

2 $$\left[ {Y = \beta_{0} + \mathop \sum \limits_{i = 1}^{3} \beta_{i} x_{i} + \mathop \sum \limits_{i = 1}^{3} \beta_{ii} x_{i}^{2} + \mathop \sum \limits_{i = 1}^{2} \mathop \sum \limits_{j = i + 1}^{3} \beta_{ij} x_{i} x_{j} + \varepsilon } \right]$$

where *Y* is the predicted response, *β*_0_ is constant, *β*_*i*_, *β*_*ii*_ and *β*_*ij*_ represent the regression coefficients for the response surface model, *x*_*i*_ and *x*_*j*_ represent the independent variables and ε is the residual associated to the experiments [46]. Only non-significant (*p* < 0.05) values were involved in constructing a reduced model, while significant (*p* > 0.05) values were eliminated.

### Verification of the models

In order to assess the adequacy of the constructed model, some random extractions were prepared to validate the model predictions. Actual values were compared with the predicted values to check the adequacy of the final reduced models. The percentage of the residual standard error (RSE) was calculated for each response.

## Results and discussion

### Model fitting and analysis of variance

RSM was employed with CCRD to investigate the effects of extraction temperature, extraction time and solvent ratio on the extraction yield, DPPH radical scavenging activity and total phenolic content (TPC) of the *C. nutans* leaves. Table 2 presents the design matrices of the actual experiments using CCRD and the predicted data for the response variables. The actual values of the response variables; extraction yields, DPPH scavenging activity, and TPC of *C. nutans* varied from 14.69–24.50% of dry weight, 46.08–80.22% inhibition and 72.25–136.00 mg GAE/g of the extracts, respectively.

**Table 2:** Design matrices of actual and predicted values of extraction temperatures (A), extraction times (B) and solvent ratios (water: ethanol) (C) for the extraction conditions of *C. nutans* leaves using the CCRD design

Run	Type	Independent variables			Code level			Response variable					
		A (°C)	B (min)	C (v/v%)	A	B	C	Extraction yield (weight %)		DPPH radical scavenging activity (inhibition %)		Total phenolic content (mg GAE/g extract)	
								Act.	Pred.	Act.	Pred.	Act.	Pred.
1	Factorial	60	80	70:30	−1	−1	−1	16.37	16.36	72.79	72.77	116.94	116.75
2	Factorial	80	80	70:30	1	−1	−1	24.50	24.48	69.87	69.86	121.63	121.99
3	Factorial	60	120	70:30	−1	1	−1	22.47	22.46	78.80	78.70	115.25	115.58
4	Factorial	80	120	70:30	1	1	−1	17.23	17.26	54.43	54.52	99.50	102.08
5	Factorial	60	80	90:10	−1	−1	1	20.68	20.64	79.43	79.32	106.13	106.28
6	Factorial	80	80	90:10	1	−1	1	23.26	22.02	69.45	69.54	103.00	102.83
7	Factorial	60	120	90:10	−1	1	1	23.51	23.53	72.95	72.95	129.75	129.55
8	Factorial	80	120	90:10	1	1	1	23.18	11.60	75.77	41.90	107.00	107.35
9	Axial	53.18	100	80:20	−1.68	0	0	22.15	22.16	74.41	74.54	111.00	111.02
10	Axial	86.82	100	80:20	1.68	0	0	18.97	18.96	46.08	45.98	97.00	96.76
11	Axial	70	66.36	80:20	0	−1.68	0	18.25	18.29	80.22	80.24	117.00	116.98
12	Axial	70	133.64	80:20	0	1.68	0	14.69	14.65	57.94	61.98	120.00	119.80
13	Axial	70	100	63.18:36.82	0	0	−1.68	17.23	23.02	74.70	74.72	119.50	119.27
14	Axial	70	100	96.82:3.18	0	0	1.68	21.85	21.86	69.60	69.61	72.25	114.90
15	Center	70	100	80:20	0	0	0	20.29	20.33	56.88	74.41	107.13	118.39
16	Center	70	100	80:20	0	0	0	21.29	20.33	74.35	74.41	117.69	118.39
17	Center	70	100	80:20	0	0	0	21.07	20.33	74.00	74.41	136.00	118.39
18	Center	70	100	80:20	0	0	0	22.44	20.33	73.53	74.41	102.75	118.39
19	Center	70	100	80:20	0	0	0	19.54	20.33	74.55	74.41	109.31	118.39
20	Center	70	100	80:20	0	0	0	20.37	20.33	74.34	74.41	119.06	118.39

**Fig. 1:**
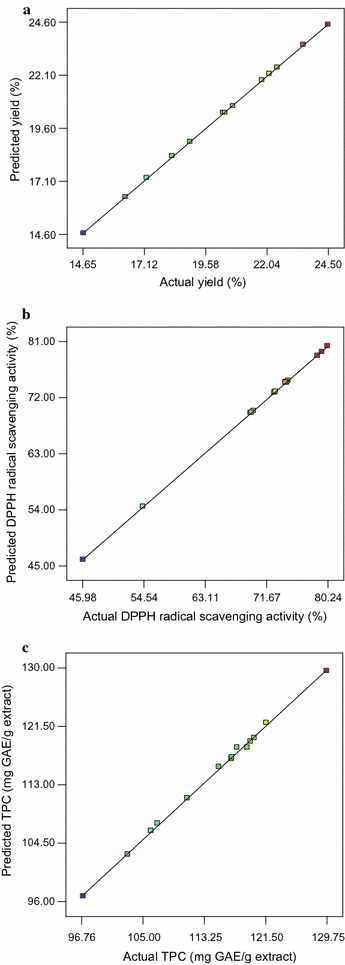
Comparison between predicted and actual values of the response variables **a** extraction yield **b** DPPH radical scavenging activity and **c** TPC of *C. nutans* leaves

By applying multiple regression analysis on the actual data, models for each of the three responses were expressed by the following quadratic polynomial model as shown in Eqs. 3–5 (Table 3). The generated equations demonstrated the empirical relationship between the dependent and independent variables for each response. A statistical method based on ANOVA was used to obtain the coefficient of determination (R^2^) for the extraction yields, DPPH scavenging activity and TPC responses which were 0.9999, 0.9999, and 0.9983, respectively. According to Jumbri et al. [47] and Hamzaoui et al. [48], a good fit with high correlation is achieved if the regression model has an R^2^ value of above 0.9. The R^2^ values obtained indicated that more than 99% of the response variables (extraction yields, DPPH scavenging activity and TPC) could be described by the RSM model. The high values of R^2^ for each response indicated that the CCRD design fitted well into the quadratic polynomial models that were developed. These results confirmed the predictability of the models in determining the optimum conditions needed to obtain the highest antioxidant activity and maximum extraction yields of the *C. nutans* leaves extracts (Fig. 1).

**Table 3:** Quadratic polynomial equations for the three responses in terms of coded factors

Responses	Equations	
Extraction yield	$Y = 20.33 - 0.95A - 1.08B - 0.35C - 3.33AB - 1.68AC - 0.80BC + 0.082A^{2} - 1.36B^{2} + 0.75C^{2}$	(3)
DPPH radical scavenging activity	$Y = 74.41 - 8.49A - 5.43B - 1.52C - 5.32AB - 1.72AC - 3.08BC - 5.01A^{2} - 1.17B^{2} - 0.80C^{2}$	(4)
TPC	$Y = 118.39 - 4.24A + 0.84B - 1.30C - 4.69AB - 2.17AC + 6.11BC - 5.13A^{2} - 0.0027B^{2} - 0.46C^{2}$	(5)

In these equations, *Y* is the predicted response, *A*, *B* and *C* are the values of the independent variables, extraction temperature (°C), extraction time (min) and solvent ratio (water: ethanol) (v/v%), respectively

Table 4 represents the regression analysis and ANOVA employed in the model fitting design in order to examine the statistical significance of the terms for all the responses. A number of runs in each response; extraction yields (6, 8, 13, 16, 17, 18 and 19), DPPH scavenging activities (8, 12, 15, 17, and 18) and TPC (4, 14, 15, 17, 18, and 19) were defined as missing independent variables (outliers) and were therefore not applied in the model design. The *F* values of 2923.40, 7138.07 and 267.02 for extraction yields, DPPH scavenging activity, and TPC respectively, indicated that all the models were significant. There was only a 0.01% chance that the values could be attributed to noise. The probability (*p* value) was relatively low in all the model responses (<0.0001), which was less than 0.05, indicating the significance of the models. A large *F* value and small *p* value is indicative that the independent variables have a significant impact on the respective response variables [49]. ANOVA revealed that all the independent variables had a significant effect (*p* < 0.05) on all responses. The extraction temperature had the most significant effect on all the responses (*p* < 0.0001). This was followed by the extraction time which had a significant value of *p* < 0.0001 towards both extraction yields and DPPH scavenging activity whereas a value of *p* = 0.0115 was obtained for TPC. Likewise, solvent ratio exhibited significant effects on DPPH scavenging activity (*p* < 0.0001), extraction yields (*p* = 0.0008) and TPC (*p* = 0.0051).

**Table 4:** ANOVA for the quadratic polynomial models developed for the response variables; extraction yields, DPPH radical scavenging activity and TPC of *C. nutans* leaves

Extraction yield (weight %)						DPPH radical scavenging activity (inhibition %)					TPC (mg GAE/g extract)				
Source	Sum of squares	DF	Mean square	*F* value	*p* value	Sum of squares	DF	Mean square	*F* value	*p* value	Sum of squares	DF	Mean square	*F* value	*p* value
Model	100.54	9	11.17	2923.40	<0.0001	1185.13	9	131.68	7138.07	<0.0001	957.55	9	106.39	267.02	<0.0001
A-extraction temperature	8.28	1	8.28	2166.64	<0.0001	750.41	1	750.41	40,677.85	<0.0001	200.12	1	200.12	502.24	<0.0001
B-extraction time	11.29	1	11.29	2954.88	<0.0001	156.47	1	156.47	8481.93	<0.0001	7.81	1	7.81	19.60	0.0115
C-solvent ratio	0.72	1	0.72	187.46	0.0008	24.00	1	24.00	1301.24	<0.0001	12.34	1	12.34	30.97	0.0051
AB	51.89	1	51.89	13,579.03	<0.0001	147.54	1	147.54	7997.49	<0.0001	126.61	1	126.61	317.75	<0.0001
AC	12.22	1	12.22	3198.83	<0.0001	15.41	1	15.41	835.51	<0.0001	27.26	1	27.26	68.41	0.0012
BC	3.01	1	3.01	787.41	<0.0001	49.40	1	49.40	2678.06	<0.0001	215.12	1	215.12	539.88	<0.0001
A^2^	0.055	1	0.055	14.52	0.0318	269.62	1	269.62	14,615.39	<0.0001	226.83	1	226.83	569.26	<0.0001
B^2^	15.45	1	15.45	4042.51	<0.0001	7.77	1	7.77	421.14	<0.0001	0.00006	1	0.00006	0.00016	0.9905
C^2^	3.59	1	3.59	938.88	<0.0001	6.81	1	6.81	369.24	<0.0001	1.37	1	1.37	3.45	0.1368
Residual	0.011	3	0.0038			0.092	5	0.018			1.59	1	0.40		
Lack of fit	0.0083	2	0.0041	1.29	0.5283	0.064	3	0.021	1.53	0.4192	0.65	3	0.22	0.23	0.8721
Pure error	0.0032	1	0.0032			0.028	2	0.014			0.94	1	0.94		
Cor total	100.55	12				1185.22	14				959.15	13			
R^2^	0.9999					0.9999					0.9983				
Adj. R^2^	0.9995					0.9998					0.9946				
Pred. R^2^	0.9975					0.9991					0.9826				
Adequate precision	181.223					308.944					61.461				

*DF* is the degree of freedom, *A* is the extraction temperature, *B* is the extraction time, *C* is the solvent ratio (water: ethanol)

The predicted R-square (Pre. R^2^) value indicates how well a regression model predicts response values; while the adjusted R-square (Adj. R^2^) indicates the descriptive power of the regression models while including the diverse numbers of variables. Every variable added to a model will increase the R^2^ value, regardless of statistical significance. Therefore, considering the Adj. R^2^ value is important to evaluate the adequacy of the model because the value tally only increases if the variables enhance the model beyond what would normally be obtained by probability. According to Koocheki et al. [50], Adj. R^2^ values above 0.9 may be used to indicate the adequacy of the model. Furthermore, a difference of less than 0.2 between Adj. R^2^ and Pre. R^2^ demonstrates the effectiveness of the model. In this study, the Adj. R^2^ values were found to be 0.9995, 0.9998 and 0.9946 for extraction yields, DPPH scavenging activity, and TPC of *C. nutans* respectively and thus, the difference in values of Adj. R^2^ and Pre. R^2^ for all the responses was less than 0.2.

The validity of the models was also confirmed using the Lack of Fit analysis, where an insignificant *p* value of more than 0.05 was indicative that the model could accurately fit with the actual data [51]. The results of this study showed that the lack of fit *p* value for extraction yields, DPPH scavenging activity and TPC were 0.5283, 0.4192 and 0.8721, respectively, indicating that all the developed quadratic polynomial models were reliable and accurate for predicting the relevant responses.

### Effects of the parameters

**Fig. 2:**
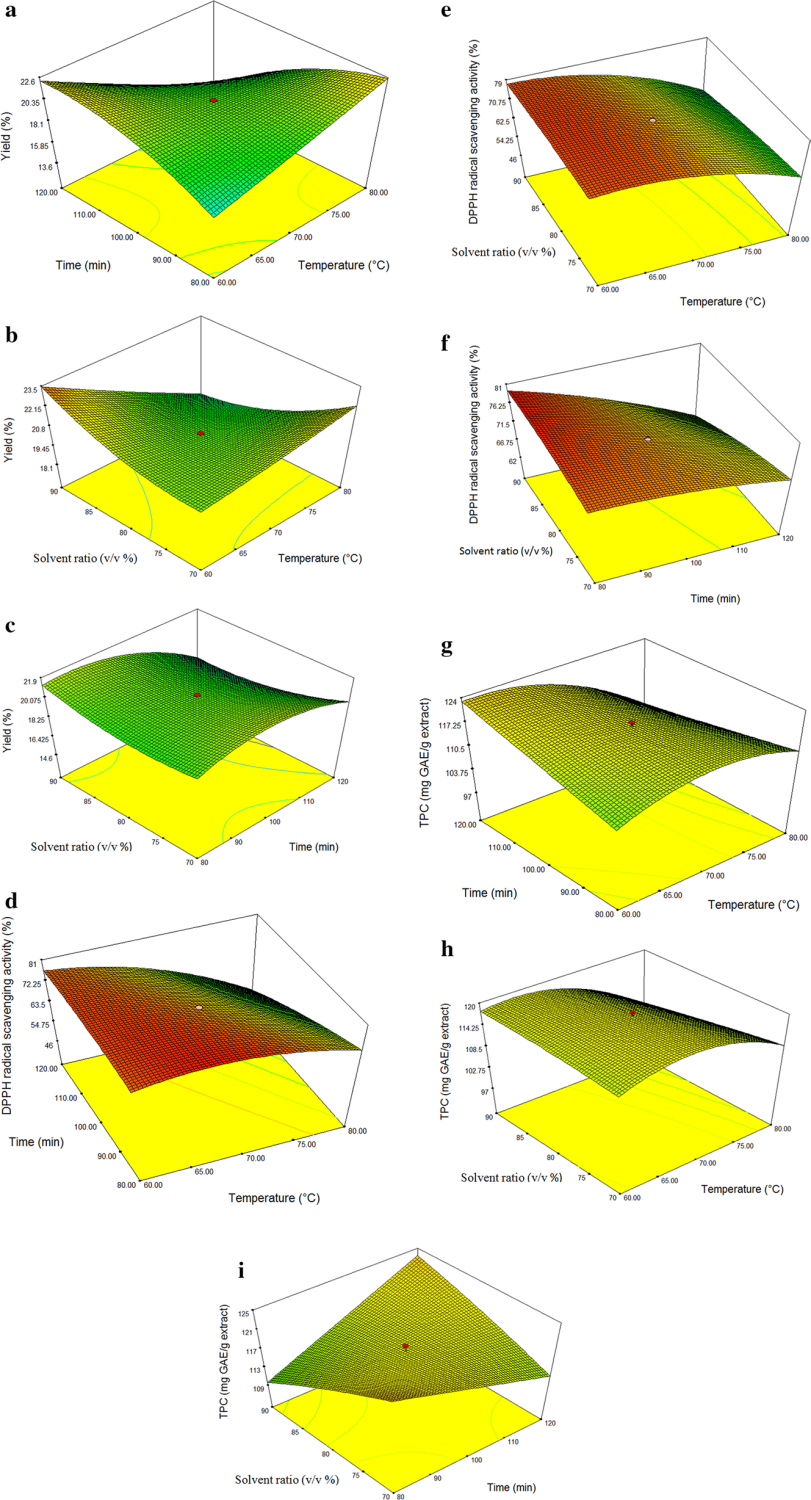
Response surface plots;** a**–**c** the interaction effect of extraction yield as a function of extraction temperature, extraction time and solvent ratio, **d**–**f** the interaction effect of DPPH radical scavenging activity as a function of extraction temperature, extraction time and solvent ratio and **g**–**i** the interaction effect of TPC as a function of extraction temperature, extraction time and solvent ratio

As shown in Fig. 2, extraction times, extraction temperatures and solvent ratios were interpreted in the ranges of 80–120 min, 60–80 °C and 70:30–90:10 (water: ethanol), respectively. The confidence interval for each response was 95% in the mentioned ranges on the plots. At a constant water to ethanol ratio (80:20), the extraction yield was found to be the highest under two conditions; a maximum temperature of 80 °C at a minimum time of 80 min and a minimum temperature of 60 °C at a maximum time of 120 min (Fig. 2a). Theoretically, under high temperatures, plant tissues are softened and the weak interactions affect the cell membranes. As a result, phenolic compounds can be easily extracted into the solvent [52]. However, a prolonged extraction time at 80 °C decreases the extraction yield because the high temperature causes the oxidation and degradation of the desired compounds [53, 54]. Conversely, by keeping the temperature at a minimum level (60 °C) for a maximum extraction time period of 120 min produced the highest yields. Hence, a prolonged exposure of the sample in the solvent, allowed sufficient time for the desired compounds to migrate into the solvent.

Figure 2b represents the effect of extraction temperatures and solvent ratios on the extraction yields. The response surface plot was generated with an extraction time fixed at 100 min. The highest yield (23.5%) was obtained at a solvent ratio of 90:10 (water: ethanol) at 60 °C. Increasing the water content in the solvent system caused swelling in the plant material which resulted in increased contact between the plant matrix and the solvent, thus contributing to an increased yield [36]. However, increasing the temperature to 80 °C significantly decreased the yield since the compounds are heat-sensitive. In contrast, at a similar temperature (80 °C) using a different solvent system (70:30), greater yields were obtained. Thus, the extracted compounds from *C. nutans* leaves could be classified into two dominant groups: the polar, water-rich compounds which were heat sensitive, and the less polar compounds that could tolerate high temperatures.

Figure 2c illustrates the effect of solvent ratios and extraction times on the yields. At a fixed temperature of 70 °C, an increase in extraction time slightly decreased the yield. The highest yield was approximately 21.9% at a solvent ratio of 90:10 (water: ethanol) and an extraction time of 80 min. Solvent ratios alone had little effect on the yield.

Figure 2d shows the interaction between extraction times and temperatures on DPPH radical scavenging activity. The lowest percentage of DPPH radical scavenging activity was observed at extraction conditions of 80 °C and 120 min at a fixed solvent ratio of 80:20 (water: ethanol). Similar observations were noted in Fig. 2a, g, where long exposure times of the samples at high temperatures produced lower yields. This could be due to the decomposition of the antioxidant compounds associated with the phenolic compounds. The lowest total phenolic content was attained under high heat (Fig. 2g). Most phenolic compounds are heat-sensitive and easily oxidized [55, 56], hence a upper limit temperature must be observed to preserve its useful components. At a similar extraction time of 120 min but with a minimum extraction temperature of 60 °C, DPPH radical scavenging activity was observed to be greater (72.25%). A decrease in extraction time had little effect on the DPPH radical scavenging activity. A similar trend was observed in Fig. 2e, where DPPH radical scavenging activity was not affected by the solvent ratio if the extraction process was conducted at the same temperature (60 °C).

DPPH radical scavenging activity under different solvent ratios and extraction times at a constant temperature of 70 °C is presented in Fig. 2f. The lowest percentage of DPPH radical scavenging activity was obtained at a solvent ratio of 90:10 (water: ethanol) using a prolonged extraction time of 120 min. As the extraction time decreased, the DPPH radical scavenging activity was greatly increased until the highest activity was reached, at above 76.25% using the same solvent ratio (90:10) but with a minimum extraction time of 80 min. Decreasing the water ratio to 70:30 (water: ethanol) led to a slight decrease in the DPPH radical scavenging activity. According to Saito and Kawabata [57] and Sharma and Bhat [58], in addition to pH and the chemical structure of the radical scavenger, DPPH radical scavenging activity could also be influenced by the polarity of the reaction medium. A water-rich solvent system (90:10) increased the antioxidant activity, which suggested that the samples were rich in antioxidant compounds.

The effect of solvent ratios and temperatures on the TPC is shown in Fig. 2h. In the beginning, lower extraction temperatures of approximately 60–65 °C had little effect on the TPC values when the solvent ratio was increased. However, above 65 °C, the TPC value decreased significantly when using a solvent system with the highest polarity (90:10). Similar observations were recorded in Fig. 2a, g, and this can be attributed to the heat-sensitive properties of some phenolic compounds.

Figure 2i depicts the TPC values with respect to solvent ratios and extraction times at a fixed extraction temperature of 70 °C. An increase in the extraction time slightly decreased the TPC value at a solvent ratio of 70:30 (water: ethanol). However, at a solvent ratio of 90:10 (water: ethanol), the TPC value increased to 121 mg GAE/g extract per time increment. A comparison of DPPH radical scavenging activity and TPC values in Fig. 2f, i for runs conducted using a solvent ratio of 90:10 at 80 min, indicated that DPPH radical scavenging activity was at its highest while TPC value was at its lowest. It is possible that the phenolic groups had no effect on the anti-radical activity measured by the DPPH radical scavenging activity assay in the stated region but other groups of antioxidant contributors had an effect. Previous investigations on *C. nutans* have established the presence of numerous potential antioxidant constituents such as fatty acids (i.e. linoleic acid, stearic acid, oleic acid, palmitic acid, myristic acid) [14], lupeol, stigmasterol, beta-sitosterol [59], chlorophylls [1] and sulfur-containing glucosides (i.e. Clinacoside A, Clinacoside B, Clinacoside C, Cycloclinacoside A1, Cycloclinacoside A2 and Triacetylcycloclinacoside A2) [60] that could be involved in neutralizing free radical damage.

### Verification of the models

In order to determine the adequacy of the final model, three randomized validation sets were performed to verify the models (Table 5). The results were compared to predicted values by calculating the RSE percentages (Eq. 6). RSE values lower than ±5 were considered to be agreement with the predicted values. The RSE values obtained indicated no significant differences between the actual and predicted values, proving that the models were adequate.

**Table 5:** Predicted and actual response values for the verification model

Set	Extraction temperature (°C)	Extraction time (min)	Solvent ratio (water: ethanol) (v/v %)	Extraction yield (%)			DPPH radical scavenging activity (%)			Total phenolic content (mg GAE/g extract)		
				Act. value	Pred. value	RSE (%)	Act. value	Pred. value	RSE (%)	Act. value	Pred. value	RSE (%)
1	75	100	80:20	19.82	19.87	0.26	70.27	68.92	1.96	117.75	114.99	2.40
2	70	100	75:25	20.69	20.69	0	73.7	74.98	1.70	116.88	118.93	1.72
3	70	100	83:17	20.18	20.29	0.55	71.28	73.89	3.53	115.25	117.69	2.30

6 $$Residual\, standard\, error \left( \% \right) = \frac{(Actual \,value - Predicted\, value)}{Predicted\, value} \times 100$$

### Optimized conditions of the extraction parameters

Optimized conditions for the simultaneous maximum extraction yields, DPPH radical scavenging activity and TPC were determined. From CCRD analysis, the optimized conditions using an extraction temperature of 60 °C, an extraction time of 120 min, and a solvent ratio (water: ethanol) of 90:10 v/v% could produce the optimum extraction yields, DPPH radical scavenging activity and TPC of 23.51, 72.95% and 129.75 mg GAE/g extract, respectively. Table 6 shows the predicted and actual response values for the optimized conditions. Under optimum conditions, the actual responses showed that the models were in good agreement with the predicted values with RSE values of less than 0.2%.

**Table 6:** Predicted and actual response values for the optimized extraction parameters

Parameter no.	Responses	Actual value	Predicted value	RSE (%)
1	Extraction yield (%)	23.51	23.53	0.09
2	DPPH radical scavenging activity (%)	72.95	72.95	0
3	Total phenolic content (mg GAE/g extract)	129.75	129.55	0.15

The range of parameters was selected based on our preliminary studies (data is not shown). Considering the need to minimize the costs of actual production, it is reasonable to estimate the economic conditions that are required in order to allow minimum energy and solvent consumption but at the same time, achieving the desired output. Thus, the extraction conditions of the *C. nutans* leaves from this study were obtained by limiting the extraction parameters to a temperature range of 60–80 °C for 80–120 min and a water-rich ratio of water to ethanol 70:30–90:10 v/v%. Water remains the cheapest and safest, eco-friendly solvent to extract bioactive substances such as polyphenols, polysaccharides, proteins and glycosides [61]. Among these water-soluble (hydrophilic) compounds, some have shown good potential as free-radical scavengers and antioxidant agents [61]. The temperature was limited to 80 °C to preserve the useful components in the *C. nutans* leaves because above this temperature, the phenolic compounds are subject to decomposition. Although, one must bear in mind that the limitations of TPC assay include poor specificity and that antioxidant activity can be influenced by any substance that can be oxidized by the Folin reagent, not only just polyphenols [62]. There are other variations to extraction parameters that can be used for the extraction of plant extracts. Thus, the selection of parameters employed in this study was focused on hydrophilic antioxidants.

## Conclusions

This study demonstrated that RSM is an effective tool for optimizing the extraction conditions of *C. nutans* leaves and allows a better understanding of the relationship between independent variables and response variables. The model was verified statistically with ANOVA. Under the optimum conditions, the actual values were in good agreement with the predicted values as RSE values for the optimum conditions were less than 0.2%. All the independent variables had a significant effect (*p* < 0.05) on all the responses which indicated that all extraction parameters employed in this study were important in the optimization process. The R^2^ values for three responses, extraction yields, DPPH radical scavenging activity and TPC were 0.9999, 0.9999 and 0.9983 respectively, suggesting that the quadratic polynomial models developed were satisfactorily accurate to be used in analyzing the interactions of the parameters (response and independent variables). The optimum conditions generated from RSM (an extraction temperature of 60 °C, an extraction time of 120 min, and a solvent ratio (water: ethanol) of 90:10 v/v%) could be used for future upscale extractions of *C. nutans* leaves by considering the temperature, extraction time, and solvent ratio for economical evaluation. This study could be useful in the development of cosmeceutical products containing extracts of *C. nutans*.

## Abbreviations

RSM: response surface methodology
CCRD: central composite rotatable design
DPPH: 1,1-diphenyl-2-picrylhydrazyl
*C. nutans*: *Clinacanthus nutans*
Na_2_CO_3_: sodium carbonate
ANOVA: analysis of variance
R^2^: determined coefficient
Pre. R^2^: predicted R-square
Adj. R^2^: adjusted R-square
DF: degrees of freedom
A: extraction temperature
B: extraction time
C: solvent ratio (water: ethanol)
TPC: total phenolic content
GAE: gallic acid equivalent
RSE: residual standard error
